# A real-world pattern on the long-term durability of the immune response to hepatitis B vaccination among people living with HIV in China: an 8.5-year retrospective study

**DOI:** 10.3389/fpubh.2026.1901939

**Published:** 2026-08-14

**Authors:** Honghong Yang, Xiang Du, Lvlang Zhang, Tongxin Li, Pengle Guo, Jingmin Nie

**Affiliations:** 1 Division of Infectious Diseases, Chongqing Public Health Medical Center, Chongqing, China; 2 Department of Clinical Laboratory, Chongqing Public Health Medical Center, Chongqing, China; 3 Infectious Diseases Center, Guangzhou Eighth People’s Hospital, Guangzhou Medical University, Guangzhou, China

**Keywords:** dose-response, hepatitis B surface antibody, hepatitis B vaccine, immune response, people living with HIV

## Abstract

The long-term durability of the immune response after primary hepatitis B (HBV) vaccination in people living with HIV (PLWH) remains unclear. This real-world study aimed to assess HBV vaccination immunogenicity in PLWH and explore the factors influencing long-term immunogenicity. A total of 195 PLWH with baseline anti-HBs < 10 IU/mL who received ≥1 dose of Heplisav-B vaccine and underwent anti-HBs measurement at 7–9 months after the first dose were retrospectively analyzed. Longitudinal follow-up of 1.5–8.5 years was conducted to evaluate long-term trend of anti-HBs titer. The study population was predominantly male (88.2%), with a median baseline CD4 + cell count of 351.8 cells/μL, and 81.0% had undetectable HIV RNA at baseline. In total, 44 (22.5%), 38 (19.5%), 100 (51.3%), and 13 (6.7%) PLWH received 1, 2, 3, and 4 vaccine doses, respectively. The initial immune response (7–9 months post-dose 1) was dose-dependent, with rates of 77% (1 dose), 79% (2 doses), 98% (3 doses), and 85% (4 doses). Compared with the 1- and 2-dose regimens, both the 3- and 4-dose regimens induced significantly higher response rates, characterized by a predominance of high responses (HRs). Over the 8.5-year follow-up period, response rates gradually decreased across all the groups. However, the 3- and 4-dose groups reached superior durability (83 and 88% response rates at year 8.5 years post-dose 1, respectively), with median anti-HBs titers persistently >100 IU/mL. Multivariable analysis revealed the number of vaccine doses and a robust early serological response were consistent independent correlates of antibody persistence at 3.5, 5.5, and 8.5 years post-dose 1, with HIV RNA negativity additionally emerging as a key factor related to the 8.5-year outcome. These findings suggest that a complete 3- or 4-dose HBV vaccination series, coupled with an early serological response, is associated with sustained long-term antibody persistence in PLWH.

## 1 Introduction

Both human immunodeficiency virus (HIV) infection and hepatitis B virus (HBV) infection are both serious worldwide public health problems. Due to their shared transmission routes, people living with HIV (PLWH) have a significantly higher risk of HBV coinfection (1). In China, the prevalence of HBV among PLWH is approximately 8–19% higher than that in the general adult population (2). HBV/HIV coinfection further increases the risk of hepatitis B chronicity, liver damage and mortality (3). Thus, clinical guidelines recommend that all PLWH who are negative for HBV antibodies should be immunized against HBV (4).

Accumulating evidence has indicated that the administration of a complete vaccine regimen is essential for achieving seroprotective antibody titers (defined as a hepatitis B surface antibody [anti-HBs] titer of >10 IU/mL) and ensuring long-term efficacy. In healthy individuals, protective antibody levels can persist for 15–20 years or longer (5, 6); however, this durable immunity is often not achieved in PLWH. Nicolini reported that only 26.9% of patients reached detectable antibodies at 84 months postvaccination (7). Consistently, a meta-analysis showed protective seropositivity in PLWH declining steeply to 38% at 2 years and 17% at 5 years (8), markedly lower than in the general population. In contrast, a retrospective Brazilian study reported a notably higher 10-year seropositivity rate of 77.1% (9), highlighting inconsistent findings across studies. Moreover, while research on HBV vaccine response among PLWH is well documented outside China, evidence on its clinical efficacy within China remains limited.

However, the completion rate of the full-course HBV vaccination (typically defined as receiving three or more doses) among PLWH remains low in China (10). This may be related to multiple interacting factors. The primary reason is suboptimal patient adherence, as some PLWH fail to fully recognize the need to complete the vaccination schedule to achieve effective and durable protective antibodies, resulting in inadequate attention to subsequent doses. High population mobility is another key factor, particularly among migrant workers and students, for whom relocation after the first or second dose frequently interrupts the vaccination course. Additionally, economic barriers, including direct vaccine costs and indirect expenses such as lost wages and transportation, further contribute to the low completion rate. Therefore, real-world data are needed on both the completion rate of HBV vaccination among PLWH in China and the post-vaccination persistence of protective antibody levels.

Regarding post-vaccination antibody persistence, evidence remains conflicting: one study found that a viral load <50 copies/mL at vaccination reduced antibody waning risk (11), while another reported contradictory results (12). Although younger age, higher CD4 + cell counts during vaccination, higher postvaccination antibody titers, and an increased vaccine dose or number of doses, are associated with prolonged anti-HBs persistence (7, 9, 12, 13), the available evidence is inconsistent. Given the limited long-term real-world evidence on HBV vaccination in PLWH, this study aimed to describe the actual completion rate and the immune response characteristics to HBV vaccination in real-world PLWH, longitudinally assess antibody persistence for up to 8.5 years, and further explore factors influencing the long-term sustainability of immune responses.

## 2 Materials and methods

### 2.1 Study subjects

This was an observational retrospective real-world study. Between October 2013 and December 2024, participants were PLWH who received primary HBV vaccination, underwent anti-HBs measurement at 7–9 months after the first dose at Chongqing Public Health Medical Center or the Guangzhou Eighth People’s Hospital of Guangzhou Medical University, and required long-term regular follow-up due to their HIV infection. The diagnosis of HIV infection was based on the Chinese Guidelines for HIV/AIDS Diagnosis and Treatment (2024 edition) (14), and all the PLWH were infected with HIV type 1. The inclusion criteria were as follows: (1) age ≥18 years; (2) baseline anti-HBs titer <10 IU/mL (anti-HBs-negative), with HBcAb-positive patients required to have undetectable (or negative) HBV DNA; (3) PLWH receiving ART; (4) have received at least one dose of HBV vaccination. The exclusion criteria were as follows: (1) pregnant or breastfeeding, (2) a history of HBV vaccination, and (3) intolerance to any component of the HBV vaccine (4) and patients for whom serological results 7–9 months after administration of the first dose of vaccine could not be obtained. Furthermore, the anti-HBs titer was measured in annually stored plasma samples up to 8.5 years after vaccination. The samples were collected during out clinic patient visits each 3–6 months.

### 2.2 Definitions

Nonresponders (NRs) were defined as those whose serum anti-HBs titers were <10 IU/mL, low responders (LRs) were defined as those whose serum anti-HBs concentrations ranged from 10–100 IU/mL, and high responders (HRs) were defined as those whose serum anti-HBs concentrations were ≥ 100 IU/mL. Analyses were stratified on the basis of the actual number of vaccine doses administered. Baseline was uniformly defined as the time point immediately before the first vaccine dose (Month 0).

### 2.3 Medical history data collection

Participants received 1 to 4 doses of recombinant hepatitis B vaccine (Kangtai Biological Products Co., Ltd., Shenzhen, China; 20 μg HBsAg/1.0 mL with aluminum hydroxide adjuvant) on schedules of 0 month (1 does), 0–1 month (2 doses), 0–1–6 months (3 doses), or 0–1–2–6 months (4 doses), with an allowable interval of ±7 days for each dose. All samples were tested retrospectively in batched runs after the completion of participant follow-up, using the same reagent lots throughout. HBV serological markers were tested by chemiluminescence immunoassays (Roche Diagnostics, Switzerland). The upper detection limit of the serum anti-HBs titer was 1,000 IU/mL. HIV RNA levels were measured by a Cobas TaqMan PCR assay (Roche Diagnostics, Switzerland; the detection limit was 20 IU/mL), and CD4 + and CD8 + T-cell counts were performed by flow cytometry (Canto II, BD, United States).

### 2.4 Statistical analysis

The demographic and clinical data were comprehensively analyzed. Statistical analyses were performed using SPSS 20.0 and R version 4.2 (lme4 package). Categorical variables are presented as frequencies (percentages), whereas continuous variables are presented as the means ± standard deviations. For multiple continuous variables, analysis of variance was employed. To compare immune response rates, the independent samples chi-square test was utilized. When the minimum expected cell count was between 1 and 5, the chi-square test with continuity correction was applied. When more than 20% of cells had an expected frequency less than 5, or when any cell had an expected frequency less than 1, Fisher’s exact test was used. Pairwise comparisons among multiple groups were performed using the Bonferroni adjustment to control for Type I errors in multiple testing. Multivariate analysis was performed using logistic regression. To analyze the trend of changes across different injection timepoints, a linear mixed-effects model analysis was conducted using the lme4 package in the R language.

## 3 Results

### 3.1 Study population characteristics

A total of 195 PLWH who received ≥1 dose of Heplisav-B vaccine and underwent anti-HBs measurement at 7–9 months after the first dose were included in the analysis. The cohort consisted of 172 males (88.2%) and 23 females (11.8%), with a mean baseline age of 31.7 ± 9.7 years. Comorbidities included hepatitis C coinfection in 3 (1.5%) patients and syphilis in 29 (14.9%) patients. All patients (100.0%) were taking antiretroviral therapy (ART), and all of whose regimens contained at least one HBV-active agent, including lamivudine (3TC), tenofovir disoproxil fumarate (TDF), or tenofovir alafenamide (TAF). The median baseline CD4 + cell count was 351.8 cells/μL, and 158 patients (81.0%) had an HIV RNA load below the detection limit at baseline. The distribution of vaccine doses received was as follows: 44 PLWH (22.5%) received one dose, 38 (19.5%) received two doses, 100 (51.3%) received three doses, and 13 (6.7%) received four doses (Table 1).

**Table 1:** The basic characteristics of 195 PLWH.

Variable	Total	One-dose (*n* = 44)	Two-dose (*n* = 38)	Three-dose (*n* = 100)	Four-dose (*n* = 13)	R/F	*p*
Sex, *n* (%)						2.101	0.564
Male	172 (88.2%)	36 (81.8%)	34 (89.5%)	90 (90.0%)	12 (92.3%)		
Female	23 (11.8%)	8 (18.2%)	4 (10.5%)	10 (10.0%)	1 (7.7%)		
Age (years)	31.7 ± 9.7	36.6 ± 11.9	30.7 ± 9.8	30.5 ± 7.9	29.9 ± 9.3	4.838	0.003
Baseline CD4 count (cells/μL)	351.8 (222.3,495.5)	342.5 (215.3,486.8)	338.0 (209.0, 472.5)	356.8 (228.5,501.0)	349.0 (221.0,493.0)	2.556	0.765
Baseline CD8 count (cells/μL)	693.5 (458.0, 931.0)	685.0 (452.5, 923.3)	679.8 (448.0,915.3)	702.5 (468.8,945.0)	695.0 (461.0,932.0)	0.601	0.812
CD4/CD8 ratio	0.52 (0.31, 0.79)	0.52 (0.31,0.78)	0.51 (0.30,0.76)	0.53 (0.32,0.80)	0.52 (0.31,0.79)	1.720	0.901
Baseline HIV RNA						4.034	0.254
Negative	158 (81.0%)	39 (88.6%)	27 (71.1%)	81 (81.0%)	11 (84.6%)		
Positive	37 (19.0%)	5 (11.4%)	11 (28.9%)	19 (19.0%)	2 (15.4%)		
Hepatitis C co-infection						1.178	1.000
Negative	192 (98.5%)	43 (97.7%)	38 (100.0%)	98 (98.0%)	13 (100.0%)		
Positive	3 (1.5%)	1 (2.3%)	0 (0%)	2 (2.0%)	0 (0%)		
Syphilis co-infection						0.351	0.965
Negative	166 (5.1%)	37 (84.1%)	32 (84.2%)	86 (86.0%)	11 (84.6%)		
Positive	29 (14.9%)	7 (15.9%)	6 (15.8%)	14 (14.0%)	2 (15.4%)		

### 3.2 Response to HBV vaccination

**Figure 1:**
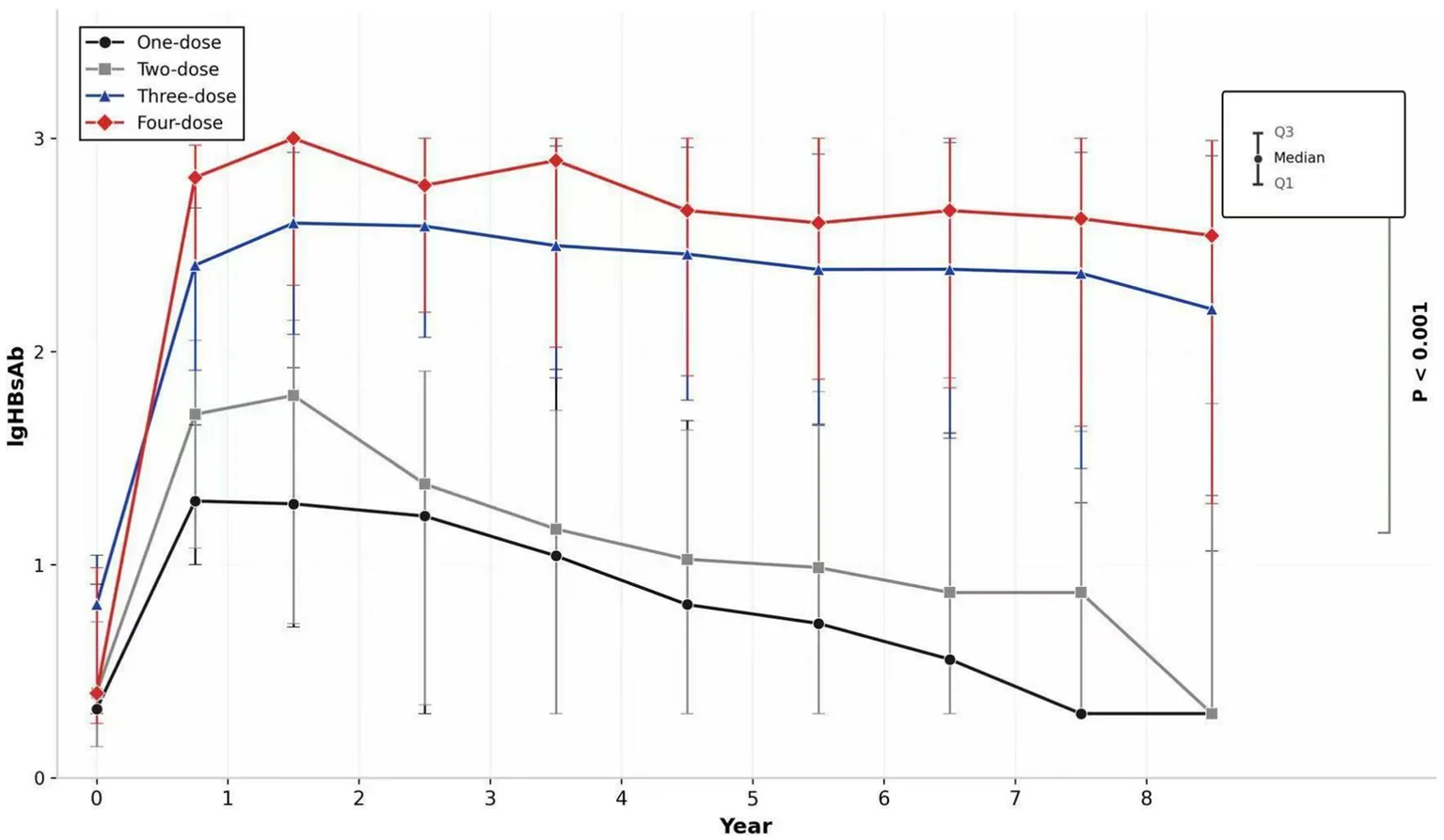
Longitudinal changes in anti-HBs titers at various time points by vaccination schedule (1- to 4-dose).

The initial immune response rates at 7–9 months post-dose 1 were 77.3% for patients who had received one dose (18.2% HRs, 59.1% LRs), 78.9% for two doses (26.3% HRs, 52.6% LRs), 98.0% for three doses (73.0% HRs, 25.0% LRs), and 84.6% for four doses (84.6% HRs, 0% LRs). Over the 8.5-year follow-up period, the response rates gradually decreased. At the 1.5 years post-dose 1, the rates were reached at 68.2% for patients who had received one dose (18.2% HRs, 50.0% LRs), 70.3% for two doses (40.6% HRs, 29.7% LRs), 94.9% for three doses (78.8% HRs, 16.1% LRs), and 92.3% for four doses (76.9% HRs, 15.4% LRs). At the 8.5 years post-dose 1, rates were reached at 26.6% for patients who had received one dose (13.3% HRs, 13.3% LRs), 45.5% for two doses (9.1% HRs, 36.4% LRs), 82.8% for three doses (56.2% HRs, 26.6% LRs), and 87.5% for four doses (62.5% HRs, 25.0% LRs) (Table 2). Compared with patients who had received one- and two-dose regimens, both those receiving the three- and four-dose regimens elicited significantly higher immune response rates (*p* < 0.001), although no statistically significant difference was observed between the three- and four-dose groups. The number of vaccine doses is a significant determinant of the immune response rate in PLWH at each monitoring point (Figure 1). Furthermore, HR predominated in the three- and four-dose groups, whereas LR was more common in the one- and two-dose groups.

**Table 2:** Immune responses to varied doses of hepatitis B vaccine in 195 PLWH.

Time points	One-dose	Two-dose	Three-dose	Four-dose	χ^2^	*p*
At 7–9 months post-dose 1 (*n* = 195)					54.195	<0.001^bcde^
NRs	10 (22.7%)	8 (21.1%)	2 (2.0%)	2 (15.4%)		
LRs	26 (59.1%)	20 (52.6%)	25 (25.0%)	0 (0%)		
HRs	8 (18.2%)	10 (26.3%)	73 (73.0%)	11 (84.6%)		
At 1.5 years post-dose 1 (*n* = 193)					52.949	<0.001^bcd^
NRs	14 (31.8%)	11 (29.7%)	5 (5.1%)	1 (7.7%)		
LRs	22 (50.0%)	11 (29.7%)	16 (16.1%)	2 (15.4%)		
HRs	8 (18.2%)	15 (40.6%)	78 (78.8%)	10 (76.9%)		
At 2.5 years post-dose 1 (*n* = 187)					57.981	<0.001^bcde^
NRs	17 (39.5%)	11 (31.4%)	6 (6.2%)	1 (7.7%)		
LRs	18 (41.9%)	16 (45.7%)	17 (17.7%)	2 (15.4%)		
HRs	8 (18.6%)	8 (22.9%)	73 (76.1%)	10 (76.9%)		
At 3.5 years post-dose 1 (*n* = 182)					56.250	<0.001^bcde^
NRs	21 (49%)	15 (45%)	7 (8%)	1 (7.7%)		
LRs	13 (30%)	12 (36%)	19 (20%)	2 (15.4%)		
HRs	9 (21%)	6 (18%)	67 (72%)	10 (76.9%)		
At 4.5 years post-dose 1 (*n* = 175)					52.227	<0.001^bcde^
NRs	21 (51.2%)	15 (48.4%)	8 (8.9%)	1 (7.7%)		
LRs	13 (31.7%)	10 (32.3%)	21 (23.3%)	2 (15.4%)		
HRs	7 (17.1%)	6 (19.3%)	61 (67.8%)	10 (76.9%)		
At 5.5 years post-dose 1 (*n* = 175)					58.219	<0.001^bcde^
NRs	23 (56.1%)	15 (50.0%)	7 (7.7%)	1 (7.7%)		
LRs	13 (31.7%)	10 (33.3%)	25 (27.5%)	3 (23.1%)		
HRs	5 (12.2%)	5 (16.7%)	59 (64.8%)	9 (69.2%)		
At 6.5 years post-dose 1 (*n* = 171)					49.870	<0.001^bcde^
NRs	23 (56.1%)	16 (55.2%)	9 (10.2%)	1 (7.7%)		
LRs	13 (31.7%)	7 (24.1%)	24 (27.3%)	4 (30.8%)		
HRs	5 (12.2%)	6 (20.7%)	55 (62.5%)	8 (61.5%)		
At 7.5 years post-dose 1 (*n* = 160)					56.545	<0.001^bcde^
NRs	26 (66.7%)	15 (55.6%)	11 (13.6%)	1 (7.7%)		
LRs	9 (23.1%)	9 (33.3%)	16 (19.7%)	4 (30.8%)		
HRs	4 (10.2%)	3 (11.1%)	54 (66.7%)	8 (61.5%)		
At 8.5 years post-dose 1 (*n* = 113)					31.770	<0.001^bcd^
NRs	22 (73.4%)	6 (54.5%)	11 (17.2%)	1 (12.5%)		
LRs	4 (13.3%)	4 (36.4%)	17 (26.6%)	2 (25.0%)		
HRs	4 (13.3%)	1 (9.1%)	36 (56.2%)	5 (62.5%)		

Statistically significant differences were found for: b. 1- vs. 3-dose; c. 1- vs. 4-dose; d. 2- vs. 3-dose; e. 2- vs. 4-dose. Pairwise comparisons were performed using the Bonferroni adjustment to control for multiple testing.

### 3.3 Longitudinal changes in anti-HBs titers

We further plotted the trends of anti-HBs titers over time for different dose groups. Anti-HBs titers peaked At 1.5 years post-dose 1 and subsequently gradually declined. Notably, although immune responses were not detected in the one- and two-dose regimen groups, the median anti-HBs titers never surpassed 100 IU/mL—failing to meet the threshold for a high responder. Furthermore, the median titers in these groups fell below 10 IU/mL within 3–4 years, indicating inadequate immunogenicity and poor response durability. In contrast, patients who completed the standard three- or four-dose group achieved high peak anti-HBs titers. Although a gradual decline was observed over time, the median titers in these groups remained above 100 IU/mL even At 8.5 years post-dose 1, demonstrating superior immune response quality and sustained long-term protection (Table 3).

**Table 3:** Anti-HBs titer levels over time by vaccination dose (1, 2, 3, or 4 doses).

Time points	One-dose	Two-dose	Three-dose	Four-dose	*χ* ^2^	*p*
Baseline (*n* = 195)	2.1 (2.0, 8.1)	2.5 (1.4, 5.4)	6.5 (2.0, 9.1)	2.5 (1.8, 9.7)	10.114	0.018^d^
At 7–9 months post-dose 1 (*n* = 195)	19.9 (10.0, 45.2)	50.8 (12.0, 113.0)	254.0 (81.6, 472.1)	655.0 (239.7, 927.8)	69.207	<0.001^bcde^
At 1.5 years post-dose 1 (*n* = 193)	19.3 (5.1, 83.8)	62.2 (5.3, 140.4)	400.6 (120.2, 860.0)	1000.0 (204.6, 1000.0)	77.552	<0.001^bcde^
At 2.5 years post-dose 1 (*n* = 187)	16.9 (2.0, 80.8)	23.9 (2.2, 80.8)	387.9 (116.8, 1000.0)	601.7 (152.9, 1000.0)	68.124	<0.001^bcde^
At 3.5 years post-dose 1 (*n* = 182)	11.0 (2.0, 82.5)	14.7 (2.0, 52.9)	313.9 (75.1, 921.1)	788.1 (104.9, 1000.0)	66.345	<0.001^bcde^
At 4.5 years post-dose 1 (*n* = 175)	6.5 (2.0, 47.5)	10.6 (2.0, 42.8)	286.7 (59.2, 907.6)	459.0 (77.0, 1000.0)	57.217	<0.001^bcde^
At 5.5 years post-dose 1 (*n* = 175)	5.3 (2.0, 45.1)	9.7 (2.0, 64.9)	242.6 (45.6, 844.1)	401.2 (74.0, 1000.0)	57.262	<0.001^bcde^
At 6.5 years post-dose 1 (*n* = 171)	3.6 (2.0, 41.5)	7.4 (2.0, 75.2)	243.3 (39.3, 957.9)	458.5 (67.5,1000.0)	52.033	<0.001^bcde^
At 7.5 years post-dose 1 (*n* = 160)	2.0 (2.0,19.4)	7.4 (2.0, 42.3)	233.0 (28.3, 859.0)	421.0 (44.6, 1000.0)	54.099	<0.001^bcde^
At 8.5 years post-dose 1 (*n* = 113)	2.0 (2.0, 11.6)	2.0 (2.0, 56.9)	158.1 (21.1, 827.8)	350.0 (19.4, 978.0)	31.903	<0.001^bcd^

Statistically significant differences were found for: b. 1- vs. 3-dose; c. 1- vs. 4-dose; d. 2- vs. 3-dose; e. 2- vs. 4-dose. Pairwise comparisons were performed using the Bonferroni adjustment to control for multiple testing.

### 3.4 Analysis of factors influencing long-term immunological responses

#### 3.4.1 Immunological response at 3.5 years post-dose 1

Univariable analysis of follow-up data at 3.5 years post-dose 1, which included variables such as sex, age, CD4 + cell count, CD8 + cell count, CD4+/CD8 + ratio, HIV RNA level, syphilis, HCV status, early immune response (at 7–9 months post-dose 1), and baseline anti-HBc status, revealed significant differences (*p* < 0.05) in the CD4 + cell count, CD4+/CD8 + ratio, HIV RNA positivity, early immune response, and baseline anti-HBc status (Table 4). Multivariable analysis indicated that the number of vaccine doses and early immune response were the factors most strongly associated with sustained immunogenicity. (Table 5).

**Table 4:** Analysis of factors influencing the immune response at 3.5 years after dose 1 in 182 PLWH.

Variable	Non responders (NRs) (*n* = 44)	Low responders (LRs) (*n* = 46)	High responders (HRs) (*n* = 92)	χ2/*t*/*Z*	*p*
Sex, *n* (%)				0.951	0.342
Male	37 (23.0%)	41 (25.4%)	83 (51.6%)		
Female	7 (33.3%)	5 (23.8%)	9 (42.9%)		
Age (years)	34.7 ± 9.8	31.1 ± 10.1	30.6 ± 9.2	2.868	0.059
CD4 count at 3.5 years post-dose 1 (cells/μL)	356.5 (248.5, 441.3)	400 (252.5, 526.3)	414.5 (340.0, 518.5)	9.141	0.010
CD8 count at 3.5 years post-dose 1 (cells/μL)	952.5 (696.5, 1180.0)	882 (625.8, 1069.0)	830 (674.8, 1073.3)	1.886	0.390
CD4/CD8 ratio at 3.5 years post-dose 1	0.36 (0.26, 0.50)	0.50 (0.33, 0.72)	0.47 (0.37, 0.65)	11.555	0.003
HIV RNA at 3.5 years post-dose 1				2.368	0.018^a^
Negative	30 (20.7%)	36 (24.8%)	79 (54.5%)		
Positive	14 (37.9%)	10 (27.0%)	13 (35.1%)		
Hepatitis C co-infection at baseline				0.569	0.569^a^
Negative	43 (24.0%)	45 (25.1%)	91 (50.9%)		
Positive	1 (33.3%)	1 (33.3%)	1 (33.3%)		
Syphilis co-infection at baseline				0.868	0.385
Negative	38 (24.8%)	40 (26.1%)	75 (49 0.1%)		
Positive	6 (20.7%)	6 (20.7%)	17 (58.6%)		
Response at 7–9 months post-dose 1				78.116	<0.001^a^
Non responders	21 (95.5%)	1 (4.5%)	0 (0%)		
Low responders	21 (31.3%)	24 (35.8%)	22 (32.9%)		
High responders	2 (2.1%)	21 (22.6%)	70 (75.3%)		
Anti-HBc Titer Levels at baseline	0.12 (0.08, 1.06)	0.16 (0.10, 0.26)	0.10 (0.08, 0.18)	7.450	0.024^a^

a Indicates that Fisher’s exact test was used.

**Table 5:** Multivariable regression analysis of long-term immune response outcomes.

Variable	Characteristic	Multivariate regression coefficient (B) (95% CI)	*p*
3.5 years after post-dose 1 (*n* = 182)	HIV RNA at 3.5 years post-dose 1	−0.816 (−1.673, 0.041)	0.062
	vaccine doses	1.227 (0.826, 1.627)	<0.001
	Response at 7–9 months post-dose 1	4.828 (2.624, 7.032)	<0.001
5.5 years after post-dose 1 (*n* = 175)	HIV RNA at 5.5 years post-dose 1	−0.841 (−1.693, 0.011)	0.053
	vaccine doses	1.306 (0.894, 1.718)	<0.001
	Response at 7–9 months post-dose 1	4.578 (2.345, 6.811)	<0.001
8.5 years after post-dose 1 (*n* = 113)	HIV RNA at 8.5 years post-dose 1	−1.239 (−2.224, −0.253)	0.014
	vaccine doses	1.209 (0.719, 1.699)	<0.001
	Response at 7–9 months post-dose 1	3.545 (1.285, 5.805)	0.002

a. Outcome variable: post-dose long-term immune response, originally categorized into three levels (NR, non-responder; LR, low responder; HR, high responder). Multinomial logistic regression was applied for this 3-level categorical outcome; unexponentiated regression coefficients (B) rather than exponentiated odds ratios are presented. b. If the three response subgroups were dichotomized into responders (LR + HR) versus non-responders (NR), binary logistic regression would be applied. If ordinal logistic regression had been adopted, the proportional odds assumption would have been assessed and reported separately. c. B = unexponentiated multivariate regression coefficient; 95% CI = 95% confidence interval for regression coefficient; P = Wald test *p*-value. d. n = number of participants at each follow-up time point.

#### 3.4.2 Immunological response at 5.5 years post-dose 1

For the 5.5-year follow-up, univariable analysis revealed a similar set of significant factors (Table 6). Multivariable analysis again revealed the number of vaccine doses and early immune response (at 7–9 months post-dose 1) were the factors most strongly associated with sustained immunogenicity (Table 5).

**Table 6:** Analysis of factors influencing the immune response at 5.5 years after dose 1 in 175 PLWH.

Variable	Non responders (NRs) (*n* = 46)	low responders (LRs) (*n* = 51)	high responders (HRs) (*n* = 78)	χ2/*t*/*Z*	*p*
Sex, *n* (%)				0.478	0.633
Male	40 (25.8%)	45 (29.0%)	70 (45.2%)		
Female	6 (30.0%)	6 (30.0%)	8 (40.0%)		
Age (years)	34.5 ± 10.8	31.9 ± 9.0	30.3 ± 9.3	2.747	0.067
CD4 count at 5.5 years post-dose 1 (cells/μL)	342.5 (245.8, 446.0)	401.0 (255.0, 511.0)	414.5 (340.0, 521.5)	9.073	0.011
CD8 count at 5.5 years post-dose 1 (cells/μL)	952.5 (696.5, 1180.0)	882.0 (625.8, 1069.0)	830.0 (674.8, 1073.3)	1.886	0.390
CD4/CD8 ratio at 5.5 years post-dose 1	0.38 (0.28, 0.50)	0.50 (0.33, 0.71)	0.50 (0.38, 0.66)	11.231	0.004
HIV RNA at 5.5 years post-dose 1				2.368	0.018
Negative	31 (22.3%)	41 (29.5%)	67 (48.2%)		
Positive	15 (41.6%)	10 (27.8%)	11 (30.6%)		
Hepatitis C co-infection at baseline				1.178	1.239^a^
Negative	45 (26.3%)	49 (28.7%)	77 (45.0%)		
Positive	1 (33.3%)	2 (66.6%)	0 (0%)		
Syphilis co-infection at baseline				1.084	0.279
Negative	40 (27.0%)	45 (30.4%)	63 (42.6%)		
Positive	6 (22.2%)	6 (22.2%)	15 (55.6%)		
Response at 7–9 months post-dose 1				67.982	<0.001^a^
Non responders	20 (95.2%)	1 (4.8%)	0 (0%)		
Low responders	21 (32.8%)	26 (40.6%)	17 (26.6%)		
High responders	5 (6%)	24 (27%)	61 (68%)		
Anti-HBc Titer Levels at baseline	0.13 (0.09, 1.01)	0.15 (0.1, 0.28)	0.1 (0.07, 0.16)	12.044	0.002

a Indicates that Fisher’s exact test was used.

#### 3.4.3 Immunological response at 8.5 years post-dose 1

At the 8.5-year follow-up, univariable analysis revealed significant differences in HIV RNA positivity, early immune response, and baseline anti-HBc status (Table 7). Multivariable analysis demonstrated that HIV RNA negativity, the number of vaccine doses, and early immune response (at 7–9 months post-dose 1) were the factors most strongly associated with sustained immunogenicity (Table 5).

**Table 7:** Analysis of factors influencing the immune response at 8.5 years after dose 1 in 113 PLWH.

Variable	Non responders (NRs) (*n* = 40)	low responders (LRs) (*n* = 27)	high responders (HRs) (*n* = 46)	χ2/*t*/*Z*	*p*
Sex, *n* (%)				0.932	0.351
Male	35 (35.4%)	21 (21.2%)	43 (43.4%)		
Female	5 (35.7%)	6 (42.9%)	3 (21.4%)		
Age (years)	34.4 ± 10.3	33.0 ± 10.3	29.7 ± 10.0	2.405	0.095
CD4 count at 8.5 years post-dose 1 (cells/μL)	384.0 (248.5, 488.0)	385.0 (276.0, 507.0)	412.0 (340.0, 524.8)	2.551	0.279
CD8 count at 8.5 years post-dose 1 (cells/μL)	933.0 (695.8, 1180.0)	861.0 (633.0, 1114.0)	834.0 (696.0, 1126.8)	0.201	0.904
CD4/CD8 ratio at 8.5 years post-dose	0.39 (0.25, 0.57)	0.5 (0.31, 0.76)	0.48 (0.39, 0.66)	3.650	0.161
HIV RNA at 8.5 years post-dose				2.426	0.015
Negative	27 (30.7%)	20 (22.7%)	41 (46.6%)		
Positive	13 (52.0%)	7 (28.0%)	5 (20.0%)		
Hepatitis C co-infection at baseline				0.920	0.421^a^
Negative	39 (35%)	26 (23%)	46 (41%)		
Positive	1 (50%)	1 (50%)	0 (0%)		
Syphilis co-infection at baseline				1.550	1.121^a^
Negative	37 (36.6%)	26 (25.7%)	38 (37.7%)		
Positive	3 (25.0%)	1 (8.3%)	8 (66.7%)		
Response at 7–9 months post-dose 1				43.321	<0.001^a^
Non responders	15 (93.8%)	1 (6.2%)	0 (0%)		
Low responders	19 (46.3%)	13 (31.7%)	9 (22.0%)		
High responders	6 (10.7%)	13 (23.2%)	37 (66.1%)		
Anti-HBc Titer Levels at baseline	0.12 (0.08, 1.56)	0.12 (0.09, 0.24)	0.09 (0.07, 0.16)	10.793	0.005

a Indicates that Fisher’s exact test was used.

#### 3.4.4 Overall longitudinal trends

Linear mixed-effects model analysis revealed that the patterns of anti-HBs decline over time differed significantly among the four dose groups (dose × time interaction, *p* < 0.001), with the 3-dose and 4-dose regimens demonstrating markedly superior durability of protective antibodies. Detailed results regarding cumulative seroprotection loss rates and multivariable analyses are provided in Supplementary Tables S1 and S2.

## 4 Discussion

In this observational retrospective real-world study of 195 PLWH followed for up to 8.5 years after hepatitis B vaccination, we found that completion of a 3- or 4-dose regimen was associated with significantly higher seroconversion rates and sustained anti-HBs titers compared with incomplete 1- or 2-dose schedules. Multivariable analysis identified completion of the full schedule and early serological response were factors significantly associated with long-term antibody persistence.

Hepatitis B vaccination is regarded as the primary and most effective primary strategy for preventing HBV infection. As such, current Chinese guidelines recommend a three-dose regimen of 20 μg recombinant HBV vaccine for adults administered at 0, 1, and 6 months (15). In clinical practice, an additional dose at 2 months is administered to a small subset of patients (16). A previous study reported that adherence to the standard HBV vaccination schedule is suboptimal among PLWH (10). In our study, only 59.2% (113/195) of PLWH completed the standard 3- or 4-standard-dose HBV vaccination regimen. Incomplete vaccination (1- or 2-dose) arose from multiple factors, including poor adherence (patients underestimated the need for a full course), high population mobility (especially migrant workers and students), economic barriers (direct and indirect costs) and so on. This relatively low completion rate underscores the challenges of maintaining adequate vaccination adherence in this vulnerable population. Our real-world data highlight the need for targeted interventions and strengthened follow-up management to improve compliance with HBV vaccination schedules among PLWH.

Prior research has indicated that the seroconversion rate observed in PLWH (17.5–72%) is markedly inferior to the rate typically achieved in the general population (90–95%) (17–19). In contrast to the findings of previous reports, our study revealed a high overall seroconversion rate among PLWH following a standard HBV vaccination schedule. The seroconversion rates were 98.0% for patients receiving the 3-standard-dose regimen and 84.6% for those receiving the 4-standard-dose regimen at 7–9 months post-dose 1, which were sustained at 94.9 and 92.3%, respectively, at 1.5 years post-dose 1. These rates are comparable to those reported in immunocompetent individuals—a finding consistent with observations by Deng et al. in a Chinese cohort (20). The high seroconversion rates observed in our study, similar to that seen in healthy populations, may be attributable to several favorable conditions present among our patients. First, patients exhibited a well-preserved immune status, as evidenced by a high median baseline CD4 + T-cell count of 351.8 cells/μL. Second, all patients were receiving ART, with 81.0% achieving virological suppression at baseline, thereby mitigating the negative impact of viral replication on vaccine response. Finally, the relatively young mean age of 31.7 years in our study population likely served as another positive factor. Moreover, most patients are not coinfected with HCV. The combination of preserved immune function, effective viral suppression, younger age, and the absence of HCV coinfection collectively explains the high seroconversion rates achieved with the standard vaccination schedule.

The seroconversion rate at 7–9 months post-dose 1 was significantly lower in groups with incomplete vaccination (77.3% for one dose and 78.9% for two doses) than in those who received three or four doses (*p* < 0.05). This difference persisted throughout the 8.5-year follow-up period. At the 8.5-year follow-up, seroconversion rates were reached in 82.8% of the 3-standard-dose group (56.2% with HRs) and 87.5% of the 4-standard-dose group (62.5% with HRs). Post-vaccination anti-HBs titer is widely used as an immunological correlate of protection. Patients with anti-HBs > 100 IU/mL after completing the HBV vaccination schedule generally maintain protective antibody levels for a longer duration, consistent with a more durable immune memory (12, 21). Consistent with this, in our study, patients who received only one or two doses had anti-HBs titers less than 100 IU/mL at 7–9 months post-dose 1, which decreased significantly over the subsequent 8.5 years. In contrast, those who completed the 3- or 4-dose regimens achieved anti-HBs titers above 100 IU/mL at 7–9 months post-dose 1, and more than 50% of them reached anti-HBs levels above this threshold throughout the 8.5-year follow-up period. The administration of a third dose was associated with vaccine immunogenicity, leading to significantly higher anti-HBs titers. Completion of a 3- or 4-dose series was strongly associated with improved long-term serological outcomes; however, this finding should be interpreted in the context of potential selection bias inherent to real-world practice.

Data concerning factors associated with a sustained immune response to HBV vaccination remain limited, and most previous studies in this area have been limited by relatively short follow-up periods. This study aimed to identify predictors of an 8.5-year sustained immunological response following HBV vaccination in PLWH. While earlier studies have reported factors such as age, sex, and CD4 + cell count as potential determinants of long-term response, our analysis identified that vaccination schedule completion and serological response at 7–9 months were significantly associated with long-term antibody persistence. Specifically, PLWH who completed the full vaccination course were more likely to achieve an immune response, and those who seroconverted at 7–9 months post-dose 1, reached significantly higher long-term response rates. In contrast, no significant associations were observed for sex, or CD4 + count—a novel finding derived from real-world evidence. Although there was a statistically significant difference in mean age among the four dose groups at baseline, this difference was no longer statistically significant in the follow-up analyses conducted at years 3.5, 5.5, and 8.5. This may reflect gradual sample size reduction from minor missing data and attenuation of the baseline age effect by other clinical factors over time. Whether age is a potential determinant of long-term response warrants validation in future larger-scale studies.

Furthermore, previous studies have suggested that effective suppression of the HIV viral load at the time of vaccination may be a critical determinant of long-term vaccine immunogenicity. In our cohort, the HIV-RNA level was identified as an independent risk factor for the immune response at 8.5 years post-dose 1. However, this association was not observed at the 3.5- or 5.5-year follow-up timepoints, despite *p* values approaching 0.05 at those intervals. Therefore, whether HIV-RNA load consistently influences long-term vaccine-induced immunity requires further investigation.

Our study has several limitations. First, the cohort mainly included PLWH with relatively preserved immune status and high CD4 + cell counts, which may limit the generalizability of the findings to individuals with advanced immunosuppression. Second, some clinical variables, including nadir CD4 + count counts and prior HIV-related history, were unavailable because part of the cohort had initiated ART at external institutions before referral. Third, the relatively small sample size in the four-dose group and the decreasing sample size during long-term follow-up may have affected statistical precision. Four, attrition from 195 to 113 at 8.5 years (58.0% retention) may have introduced selection bias if completers differed from those lost, but reasons for missingness were unavailable due to the retrospective design, precluding sensitivity analyses. Fifth, breakthrough acute hepatitis B infections in participants with low immune responses were not systematically recorded, so acute infection rates could not be estimated, therefore, our findings regarding anti-HBs persistence should be interpreted as immunological correlates rather than direct evidence of clinical protection against HBV infection. Additionally, subclinical HBV exposure during follow-up may have boosted anti-HBs titers, potentially overestimating long-term durability. Given the funding constraints of this retrospective study, only anti-HBs testing was performed; the absence of serial anti-HBc data precluded assessment of HBV exposure or infection history during follow-up. Despite these limitations, our findings reflect real-world vaccination behaviors and support the importance of completing the full HBV vaccination schedule in PLWH.

## 5 Conclusion

Our study involved a real-world comparison of long-term antibody persistence and serological response rates over up to 8.5 years among PLWH receiving one, two, three, or four doses of the hepatitis B vaccine. A significantly higher serological response rate was observed in patients receiving three or four doses of the vaccine than in those receiving one or two doses. Our findings also suggested that the administration of a greater number of vaccine doses (specifically 3 or 4 doses), combined with the presence of a serological response at 7–9 months post-dose 1, was associated with a greater likelihood of maintaining long-term antibody persistence.

## Data Availability

The datasets presented in this article are not readily available because. The original data in this study can be obtained from the corresponding author upon reasonable request. Requests to access the datasets should be directed to yangfengzhuoyu@163.com.
