# Supplementary material for: A real-world pattern on the long-term durability of the immune response to hepatitis B vaccination among people living with HIV in China: an 8.5-year retrospective study
Source: Front Public Health. 2026 Aug 14;14:1901939. doi: 10.3389/fpubh.2026.1901939 (PMC13522117; doi:10.3389/fpubh.2026.1901939)
Supplement: Supplementary file 1 [file Supplementary_file_1.DOC]

**Supplementary Table S0. Linear Mixed-Effects Model: Fixed-Effects Results for Longitudinal anti-HBs Titer Changes**

| Fixed Effect | B | SE | t | P |
| --- | --- | --- | --- | --- |
| Intercept (Constant) | 1.514 | 0.336 | 4.499 | <0.001 |
| Vaccine dose frequency | 0.868 | 0.130 | 6.671 | <0.001 |
| Follow-up time | -0.133 | 0.035 | -3.829 | <0.001 |
| Dose x Time interaction | 0.084 | 0.013 | 6.279 | <0.001 |

**Supplementary Table S1. Cumulative Analysis of Seroprotection Loss (anti-HBs < 10 IU/mL) by Vaccine Dose Group over 8.5 Years of Follow-up**

| Group | Total N | Valid Cases at 8.5-year Follow-up (N) | Loss of Protection N | Cumulative Loss Rate (%) |
| --- | --- | --- | --- | --- |
| 1-dose | 44 | 28 | 22 | 78.6 |
| 2-dose | 38 | 11 | 6 | 54.5 |
| 3-dose | 100 | 64 | 11 | 17.2 |
| 4-dose | 13 | 10 | 1 | 10.0 |
| Total | 195 | 113 | 40 | 35.4 |
